# Hypertension and diabetes in Africa: design and implementation of a large population-based study of burden and risk factors in rural and urban Malawi

**DOI:** 10.1186/s12982-015-0039-2

**Published:** 2016-02-01

**Authors:** Amelia Catharine Crampin, Ndoliwe Kayuni, Alemayehu Amberbir, Crispin Musicha, Olivier Koole, Terence Tafatatha, Keith Branson, Jacqueline Saul, Elenaus Mwaiyeghele, Lawrence Nkhwazi, Amos Phiri, Alison Jane Price, Beatrice Mwagomba, Charles Mwansambo, Shabbar Jaffar, Moffat Joha Nyirenda

**Affiliations:** Karonga Prevention Study, Karonga, Malawi; London School of Hygiene and Tropical Medicine, London, UK; Malawi Ministry of Health, Lilongwe, Malawi

**Keywords:** Non-communicable diseases, Hypertension, Diabetes, Methods, Epidemiology, Sub-Saharan Africa

## Abstract

**Background:**

The emerging burden of cardiovascular disease and diabetes in sub-Saharan Africa threatens the gains made in health by the major international effort to combat infectious diseases. There are few data on distribution of risk factors and outcomes in the region to inform an effective public health response. A comprehensive research programme is being developed aimed at accurately documenting the burden and drivers of NCDs in urban and rural Malawi; to design and test intervention strategies. The programme includes population surveys of all people aged 18 years and above, linking individuals with newly diagnosed hypertension and diabetes to healthcare and supporting clinical services. The successes, challenges and lessons learnt from the programme to date are discussed.

**Results:**

Over 20,000 adults have been recruited in rural Karonga and urban Lilongwe. The urban population is significantly younger and wealthier than the rural population. Employed urban individuals, particularly males, give particular recruitment challenges; male participation rates were 80.3 % in the rural population and 43.6 % in urban, whilst female rates were 93.6 and 75.6 %, respectively. The study is generating high quality data on hypertension, diabetes, lipid abnormalities and risk factors.

**Conclusions:**

It is feasible to develop large scale studies that can reliably inform the public health approach to diabetes, cardiovascular disease and other NCDs in Sub-Saharan Africa. It is essential for studies to capture both rural and urban populations to address disparities in risk factors, including age structure. Innovative approaches are needed to address the specific challenge of recruiting employed urban males.

## Background

Chronic non-communicable diseases (NCDs), particularly diabetes and cardiovascular disease (CVD), are becoming leading causes of morbidity and death in Sub-Saharan Africa (SSA) [1, 2]; it is estimated that by 2030, over 18 million people in SSA will have diabetes [3], while deaths attributable to cardiovascular disease are projected to increase from 1.2 million in 2004 to 2.5 million in 2030 [4]. NCDs will strain the already stretched health systems. There is an urgent need for countries in SSA to respond effectively to these changing health needs, before the NCD epidemic threatens gains in life expectancy and socioeconomic development that have been achieved in combating infectious diseases.

The lessons learnt from the international response to HIV and successful scale-up of public health based anti-retroviral treatment (ART) programmes, despite limited infrastructure in the region, with concomitant decreases in adult mortality [5–8] hold promise for effective management of chronic NCDs, in tandem with appropriate prevention strategies. Effective prevention will require a clear understanding of the important local determinants of risk. Traditional risk factors (such as smoking, obesity, high salt intake, sedentary lifestyle and pollution) are likely to play a part in Africa as in other places but there are few data on the distribution of these factors or the strength of their associations with NCDs in this setting. Considerable variation in findings between countries, and inconsistent findings between urban and rural locations or sub-populations, have been observed in Africa, but these findings are based on a few, small studies [9–16]. Although the varying speed at which populations are undergoing the epidemiological transition may account for some of the observed differences, they are also likely to result from lack of standardisation in study design and measurement techniques.

To understand better the distribution of NCDs in SSA will require exploration of other factors that may have unique importance in the region, including potential interactions between NCDs and communicable diseases (such as HIV and its treatment), as well as understanding the relationship between malnutrition (particularly in early life) and risk of NCDs later in life [17–19].

Like other countries in SSA, Malawi is experiencing rapid increases in the prevalence of common NCDs. A recent WHO STEPS survey reported high rates of hypertension and diabetes but the sample size was small (n = 5206), age groups limited to 25–64 years, and 25 and 40 % of outcome data missing for blood pressure and fasting blood glucose, respectively [13].

The design and implementation of a large population-level cross-sectional study are presented; a study that aims to describe the burden, and determinants of hypertension, diabetes and lipid disorders in rural and urban Malawi. The study baseline data (that include questionnaire responses, biophysical measurements and biological specimens), will provide a valuable platform for future research to investigate into pathways to the development of cardio-metabolic disorders in this setting, which, in turn, will inform novel context-specific intervention studies aimed at both prevention and treatment. The lessons from this work will have wider relevance in the region both for policy and research.

In this paper we describe the process of the design and implementation of the project, and discuss the successes and challenges of our approach, to inform the interpretation of our findings and to share the lessons we have learnt with researchers and policy makers in this field.

## Design, methods and rationale

### Context

The study is part of a collaborative research programme by the Malawi Epidemiology and Intervention Research Unit (MEIRU), previously Karonga Prevention Study (KPS); a partnership between the London School of Hygiene and Tropical Medicine, the Malawi Ministry of Health (MoH) and the Malawi College of Medicine, funded through a Strategic Award from the Wellcome Trust. The key research questions were developed jointly in a stakeholders’ meeting in Lilongwe, the capital city of Malawi, attended by policy makers, policy implementers and researchers; the ideas were discussed in the context of the Malawi National Health Research Agenda [20]. The NCD research was developed as extension of a long-standing programme of medical research (focussing on Leprosy, TB and HIV) in the rural site in Karonga. The partners were consulted on selection of a new urban study site in Lilongwe, which was established to enable rural–urban comparison of findings.

### Research objectives

The high level objectives of the work, laid out in the funding proposals, were to undertake internationally leading research on the control of important non-communicable diseases relevant to Africa in partnership with the College of Medicine and the Ministry of Health, Malawi, and to develop research capacity.

More specifically we proposed to quantify the burden of specific NCDs, their risk factors and the barriers to accessing care, and to design and evaluate interventions which lead to effective NCD control in Africa by; (1) determining the burden of the major NCD risk factors—hypertension, diabetes, hyperlipidaemia, smoking, obesity, physical inactivity and alcohol, salt and saturated fat intake—and exploring how these vary between urban and rural populations, with age and gender; (2) describing the uptake of and retention in care among patients identified during screening with hypertension or diabetes and referred to clinical services; and (3) measuring the prevalence and clustering of NCD risk factors in people with HIV/AIDS (on ART or not on ART).

### Sample size

In the rural DSS, all 18,000 adults will be invited to participate. It is anticipated that by the conclusion of enumeration of the urban study site that 23,000 adults will be identified and invited. The large sample size will enable (a) calculation of precise age-sex urban/rural specific prevalence of key outcomes and risk factors and their distribution and clustering by HIV and socio-economic status; (b) examination of associations between outcomes and risk factors, for example characterising the relationships between anthropometric indices and outcomes in different age- and sex- groups and exploring urban rural differences in these relationships; (c) generation of baseline data on cohorts of a adequate size for future estimates of incidence of diabetes and hypertension; and (d) creation of cohorts of newly diagnosed diabetic and hypertensive patients and people with impaired fasting glucose or early hypertension. This will facilitate detailed future studies to investigate the natural course of disease and/or impacts of potential interventions.

### Study setting

As with most African countries, Malawi is undergoing urbanisation, but currently more than 80 % of the population live in the rural areas [21]. A rural and an urban area were included to explore the differences in lifestyle and prevalence of outcomes associated with urbanisation—the Demographic Surveillance Site (DSS) in southern Karonga (Northern Region, Malawi) as a typical rural population, and Area 25 of Lilongwe City (Central Region, Region) representing urban populations. The rural area is an established research site [22] and the urban site was considered appropriate for conducting the NCD studies, as the resident population represents a broad spectrum of socio-economic groups and the MoH planned to establish a chronic care clinic at the Area 25 health centre.

**Fig. 1:**
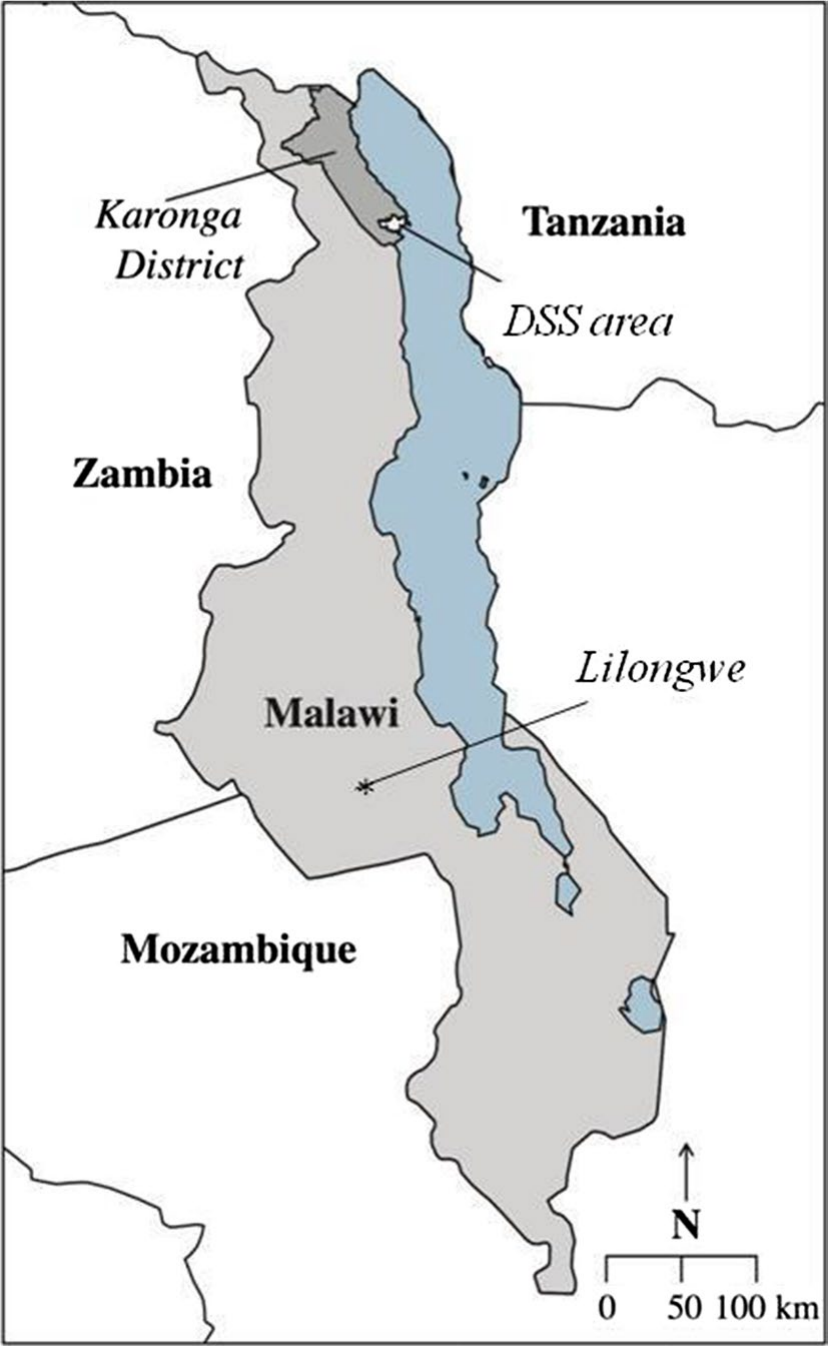
Karonga Demographic Surveillance Site and Lilongwe, Malawi

In Karonga, the study was nested in the Karonga DSS which was set up in August 2002 with a baseline census to register all individuals and households in the study area [23]. Reporting by village informants on vital events and migrations forms a continuous registration system. The DSS is located on latitude −10.43° and longitude 34.27° and is bordered by Lake Malawi in the east, Nyika National Park in the west; the north and south demarcation follows village boundaries (Fig. 1). The DSS has a population of about 36,000 individuals based on 2012 mid-year population estimates, most of whom are rural dwellers who depend on subsistence farming and fishing for their livelihoods [24] and are scattered over an area of 135 km^2^. There are two small trading centres and a port and truck depot. The DSS area is served by two hospitals: Karonga District Hospital which is 70 km from the DSS; and Chilumba Rural Hospital which along with four minor health centres, is within the surveillance site.

Lilongwe, declared the capital city of Malawi in 1975, is one of four cities in Malawi. Area 25 is a residential area located on latitude −13.95° and longitude 33.76° (Fig. 1). The area is a mixed urban population with civil servants, commercial and non-governmental organisation employees as well as students and large populations in casual and informal employment or petty trading, and seeking work. Much of the commercial employment is with tobacco processing factories and industrial estates that are located approximately 5 km from the study area. Area 25 is considered a traditional high density area, it covers land area of about 23 km^2^ hectares and reached a population of 64,650 in 2008 which rose from 39,132 in 2005 [25]. Area 25 is served by one primary government health centre and one mission health centre located within the study area and is approximately 15 km from Kamuzu Central Hospital, the main tertiary hospital in Lilongwe, and 20 km from Bwaila Hospital, which functions as a secondary referral centre.

### Study population

Although the prevalence of diabetes and hypertension are low in younger people (<25 years), all adults aged 18 years and above were invited to take part in the study, in contrast to most studies which have excluded individuals of less than 25 years old, because of expected low outcome rates. The rational for including this age group was to enable future investigations into associations between exposures experienced in younger adult life and risk for development of NCDs in later life, and for comparing trends over time in different age cohorts. An upper age restriction was not imposed, so that the total burden of diseases in this population could be described, including the urban/rural differences that might be driven by the contrasting age distributions.

All adults normally resident at a household (i.e. not a temporary member with intent to leave) in either of the study areas and able to consent were eligible to be included in the study, from May 2013 onwards. Individuals who identified themselves as visitors were excluded.

### Area demarcation

**Fig. 2:**
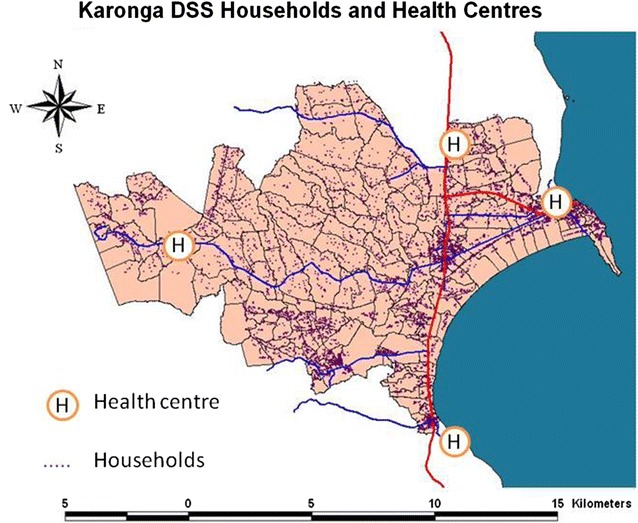
Map of Karonga Demographic Surveillance site with cluster boundaries and household density

In the Karonga DSS each village is divided for vital events reporting purposes into smaller geographical area (“clusters”) that represent an average of 35 households (Fig. 2). Groups of approximately 10 clusters in a defined geographical area are combined to form the 21 reporting groups (henceforth “study zones”), which make up the surveillance area. Detailed methods of the demographic surveillance are described elsewhere [24].

**Fig. 3:**
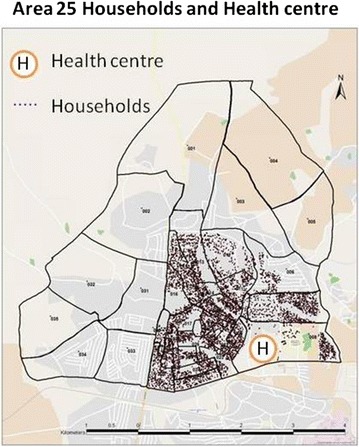
Map of Area 25, Lilongwe with enumeration area boundaries and household density of those participating to date (background © openstreet map, enumeration boundaries © Malawi National Statistics Office)

In Lilongwe, Area 25 can be sub-divided into 35 Enumeration areas (Fig. 3), demarcated by the National Statistical Office during the 2008 population and household census, which have, on average 480 households each. These were used as administrative boundaries of “study zones” for the survey.

### Stakeholder and community sensitization

Sensitization took place at district and community levels: initially with the District Health Offices and in the urban area, the police and the city council, later with traditional authorities and local head(wo)men. In both rural and urban areas periodic meetings were held, attracting between 150 and 800 people, where drama and song were used as communication tools. In the urban area a vehicle-mounted public address system was used to further inform the public prior to enumeration. Leaflets and information sheets about the study were distributed prior to the survey, introducing the partners engaged in the study and the funding body, and including information on what the participants should expect if they take part in the study. This included the meaning of “participation”, rationale for blood sampling and destination of the samples, and the risk and benefits of taking part.

### Enumeration

In Lilongwe as a new site, enumeration was conducted prior to the conduct of the survey. This process involved systematically approaching each household and identifying a suitable informant, (a resident, a domestic worker or in some cases a neighbour), to collect the required data on household membership; names and sex of household members thought to be close to 18 years old or above, and location details. Global positioning system (GPS) co-ordinates were collected using the tablet computer and every household was given a unique “household” number which is bar-coded, scanned onto the household electronic data collection form, and chalked onto a door or gate post. These enumeration data were printed out to create a hard copy register for each sector listing households, GPS co-ordinates and the name and sex of members presumed to be eligible. After the first 7 months of field work, there was a lapse between enumeration and survey, permanently resident households and individuals new to the area who had not been enumerated were added to the registers at the time of the survey.

In the rural area, a hard copy register was printed for each cluster as required, with the same information (with the addition of age), generated from up-to-date demographic surveillance data, to facilitate the field work there. Details of the DSS procedures are described elsewhere [22], but it is important to note that births and deaths are reported on a monthly basis and migrations updated annually.

### Training

The field and clinical teams recruited for the survey underwent a 15 day training programme on use of electronic data capture devices, collection of biophysical measurements, hypertension and diabetes, lifestyle counselling, blood samples collection and handling, full (or refresher) HIV counselling training and research ethics as appropriate. Consent, questionnaire and measurement procedures were tested and piloted repeatedly in workshops at both sites, initially on team members, then colleagues, then on volunteer members of the public. Pilots included blood draws and laboratory analysis. Project and government clinical and nursing staff underwent training on lifestyle counselling and management of diabetes and hypertension.

### Data collection and study procedures

The study protocol was developed through collaboration between scientists from the urban and rural site, and cross-site team visits, for all cadres of staff, were conducted for training and implementation of field work. The study was managed by a senior team who had oversight of both rural and urban sites.

Development of the study questionnaire was heavily informed by existing questionnaires including the WHO Physical activity questionnaire [26] Hyderabad [27, 28] Blantyre Health Study [29] and the STEPS survey questionnaire [13], to enable comparison with findings in other settings, as well as standard questionnaires from previous KPS studies. Anthropometric and blood pressure measurements were done to standardised protocols.

Five teams of fieldworkers (each consisting of nurse, male interviewer and female interviewer) were deployed in each site, supported by a field supervisor. The field work was managed in such a way that female field interviewers could examine female study participants and male field interviewers are could examine male study participants when requested—particularly when taking waist and hip measurements.

Android operating system tablets were used for main survey data collection using the Open Data Kit platform. Electronic data collection templates were uploaded onto the tablets at the start of the survey and each time there was a form amendment. The questionnaires were available on the tablets in English, Chichewa (the main language of the Central Region) and Chitumbuka (the main language of the Northern Region), with the facility to toggle between the three languages at any time. Range checks and prompts were programmed into the forms. The tablets were charged overnight for field work with the battery lasting two working days. The tablets have integral GPS receivers which enable the fieldworkers to take co-ordinates. The tablets were also used to scan identification barcodes stickers. Specimen linking and other supporting forms were paper-based. These were double-entered in Access databases, verified and amended where necessary. A paper consent form record was also held.

Attempts were made to make contact with each individual listed in a household. It was recorded in the register if individuals in either area had left permanently, died, were ineligible (due to being under 18 or unable to give informed consent) or had already been included in the survey in a prior location. If individuals were not found at home, the household was revisited up to three times before the individual was recorded as “missed” in the register. “Bookings” were made through household members present to try to contact absent members. In the urban area, after the first 2 months of the survey, weekend working was instituted and at least one weekend visit had to be conducted before an individual could be marked as “missed”. Evening recruitment was considered, but not implemented due to security concerns. In a sample of urban enumeration areas, the approximate age of missed or refusing individuals was determined by interview of an informant or (for those refusing) the estimation of the interviewer in order to estimate the population age distribution and age-sex-specific participation rates. For those individuals found at home, informed consent was sought from all adults aged 18 and above residing in the study areas at the time of the main survey. For each person found a study identification number was assigned, whether or not the person consented. Each individual could consent separately for the main survey, the venepuncture and any sub-studies, as appropriate. For those that gave informed consent to take part in the survey, a questionnaire was administered including individual and household variables.

Anthropometric measurements; weight, height, waist circumference, hip circumference and mid upper arm circumference, were taken. After 30 min of inactivity, three blood pressure measurements, with 5 min resting time in between, were collected using portable electronic devices (OMRON Healthcare Co., Ltd. HEM-7211-E, Model M6), and the average of the last two readings was used, to allow for the phenomenon of raised blood pressure following activity or during unfamiliar procedures. Suspected hypertension was defined as systolic blood pressure of greater than or equal to 140 mm Hg and/or diastolic blood pressure of greater than or equal to 90 mmHg, or on regular antihypertensive medication. Participants were issued with a “health passport” (patient-held record used for outpatient clinical details in Malawi) if they did not already have one.

The participants were advised to fast overnight, for a minimum of 8 h, and the nurses returned the following morning (usually between 5 am and 8.30 am) to collect fasting blood samples and offer HIV screening using a rapid test. Diabetes was defined as fasting blood glucose of greater than or equal to 7.0 mmol/L, or on regular medication for diabetes, or a previous self-reported diagnosis of diabetes. Participants who had not fasted were re-visited once; if a fasting sample was not available the second time, then a non-fasted sample, together with details of the time of the last meal, was taken.

### Laboratory procedures

Formal laboratory tests on fasting samples, were used to determine blood glucose (FBG) and lipid profile from fasting blood samples. HbA1c was performed in a sample of individuals to investigate its utility as a diagnostic tool for diagnosis of diabetes in this population. Samples were batched daily and transferred to the respective laboratories the same morning. At each site, all assays were undertaken using two automated analysers (Beckman Coulter Chemistry analyzer, Model: AU480). The laboratories participate in an external quality control scheme (Thistle, RSA) and internal controls were conducted, exchanging samples between the two sites for repeat testing to ensure consistency of measurements.

### Referral process and home visits

Results of blood pressure screening and body mass index were recorded in the participant’s Health Passport. Overweight and obese study participants were counselled by the field staff on lifestyle modification. Study participants were referred to the government health institution within each of the sites, for confirmation of diagnosis and/or treatment when any abnormal health conditions (hypertension, diabetes, HIV) are identified, by means of a referral slip. Participants with suspected hypertension were notified and referred during the face-to-face interview process and were asked to wait for 3 days for any abnormal laboratory results before going to the clinic, unless hypertension was severe, in which case an urgent referral was made. Abnormal blood results were reported directly to the participants at home, and recorded in their health passports. Blood results within normal range were made available at the clinics and, in the urban area, delivered to the household on request.

All study participants who did not report to the clinic within 30 days of being referred, were visited at their home to ascertain reasons for not taking up referral.

### Management of hypertension and diabetes

In the urban site, Area 25 health facility had been flagged for establishment of a chronic care NCD clinic to relieve pressure on the central hospital outpatient clinic. This clinic was enhanced by deployment of a study clinical officer, a clinic clerk and nursing staff. In the rural health facility the same level of support was supplied to manage the increased demand for clinical consultations that had been anticipated as a result of the survey.

The study staff operated within the parameters of the MoH clinics, whilst logging and documenting attendances carefully. A study clerk received the patients at each of the clinics, and procedures were in place to identify study participants who omit to bring their referral slips. Donations of drugs were secured from CIPLA (first and second line oral formulations) for hypertension and diabetes, sufficient to supplement those available through MoH services and to ensure uninterrupted supply. The clinical staff managed referred survey participants and non-participants according to standard hypertension and diabetes treatment algorithms which are based on national treatment guidelines [30].

There is a commitment to continuing this service for 12 months after the completion of the survey prior to handover to MoH teams.

### Data linkage and management

Each participant was given a unique study number that linked all the data collected on them in the survey, in each sub-study and laboratory results. A study identification number sticker was also placed in their “Health Passport” and on a referral slip if they were referred for care. In both urban and rural sites [22], spousal and household data linkages were created and stored. In the urban site health system electronic identity numbers [31, 32] were also collected, where available, from stickers on individuals’ health passports.

Patients attending for clinical care were registered using their study identification number.

### Data management

After each data collection shift, the data were uploaded from the tablets into a MySQL database on a local project server. Check programs were then used to detect any problems such as duplicate identifiers. Clean data were then transferred into the main project MS Access database, also on the local project server. Original records were kept as an audit in a MySQL archive database. Daily, weekly and monthly backups were created and held off-site on secure servers.

Currently data are available only to study scientific staff and approved collaborators. As part of a major initiative to make programme data more accessible to regional scientists in training and the wider research community to maximise public health benefit, and through direct support from the Wellcome Trust, a major collection of these data will be made available in a suitably anonymised form on an open data platform after the primary analyses have been completed.

### Analysis of participation data

The enumeration or demographic surveillance datasets were merged with the study questionnaire, physical examination and laboratory result data and analysed using Stata 13. Data on missed participants from sampled enumeration areas were combined with participation data and demographic surveillance data to create age and sex categories for creating population distributions. Hypertension, diabetes, body mass index, alcohol use, smoking and household possession variables were used to explore the differences between participants who were found easily and those who were difficult to reach, by using logistic regression and adjusting for age and sex.

### Ethical approval

Ethical approval for the study was granted by the Malawi National Health Sciences Research Committee (NHRSC) protocol number #1072, and London School of Hygiene and Tropical Medicine (LSHTM) Ethics Committee protocol number #6303.

## Results

### Timescale

Data collection started in June 2013 and is ongoing at the time of this analysis. It is expected that the survey will be completed by July 2015.

In the urban area, enumeration was started 4 months in advance of the survey team, to allow full piloting of the enumeration procedures. Some areas enumerated were not included (on the rural periphery of the area) as the population was very scattered and was found to include farming communities. More recently, the survey took place immediately after the enumeration procedures. Time from enumeration to survey varied from 1 week to 11 months.

### Participation rates in survey

**Fig. 4:**
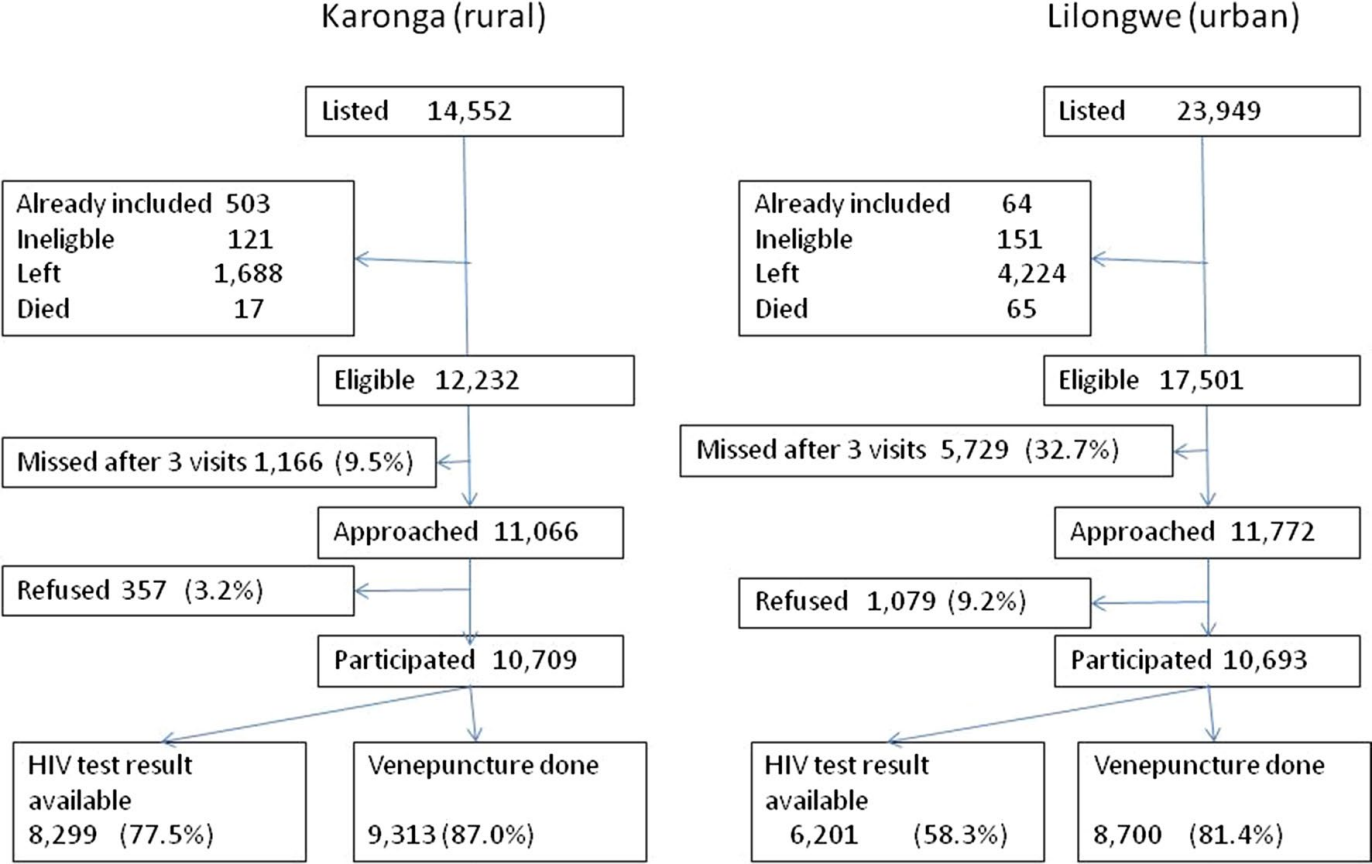
Recruitment and participation; rural and urban sites

Data on participation are presented for the first 16 (of 21) study zones (regularly updated by demographic surveillance) to be completed in the rural area and the first 23 (of 35) study zones in the urban area. This represented 14,552 and 22,005 potential participants in rural and urban sites, respectively. However, only 11,066 individuals (from 6184 households) and 11,772 individuals (from 6619 households in the rural and urban areas (respectively) were approached. The rest were not found at home after three visits (1116 and 5729 respectively), had previously participated at another household, were ineligible, had died or had left permanently (Fig. 4).

### Ease of finding on first visit

In the rural area, of those found and asked to consent, 74.3 % were found on the first visit to the household, 19.7 % on the second visit and 6 % on the third. By study zone, the proportion found on first visit varied from 65 to 82 %. In the urban area, 77.8 % of participants were found on first visit, 16.1 % on the second and 5.9 % on the third. This varied across enumeration areas from 63.9 to 89.0 %. When stratified by site, there was no difference in the age distribution of those found on first, second or third visits although women were more likely to be found on a first visit than men.

In the urban area, 18.7 % of interviews with women and 28.0 % of interviews with men were undertaken at the weekend as the participants could not be found during the week.

### Acceptance of different elements of the study

96.7 % (10,709/11,066) of those approached in the rural are accepted to take part in at least one component of the study, compared to 91.8 % (10,693/11,772) in the urban area. All participants who consented to the interview were willing to have anthropometric and blood pressure measurements performed and all but 13 individuals answered questions about HIV testing history and ART status. In Karonga, 77.5 % of participants either consented to HIV testing or had consented to be tested in another recent study (Fig. 4). In Lilongwe testing was accepted by 58 % of participants. In Karonga 7.2 % of those who did not accept rapid testing self-reported to be HIV-positive, 15.9 % in Lilongwe. The acceptance rates for next-day biochemical tests; venepuncture collection for fasting glucose and lipid were in excess of 90 % at both sites although some who consented did not keep their next-day appointment (~5 %) (Fig. 4). More than 99.5 % of samples were collected from individuals who had reported to have fasted according to instructions.

### Population characteristics

**Fig. 5:**
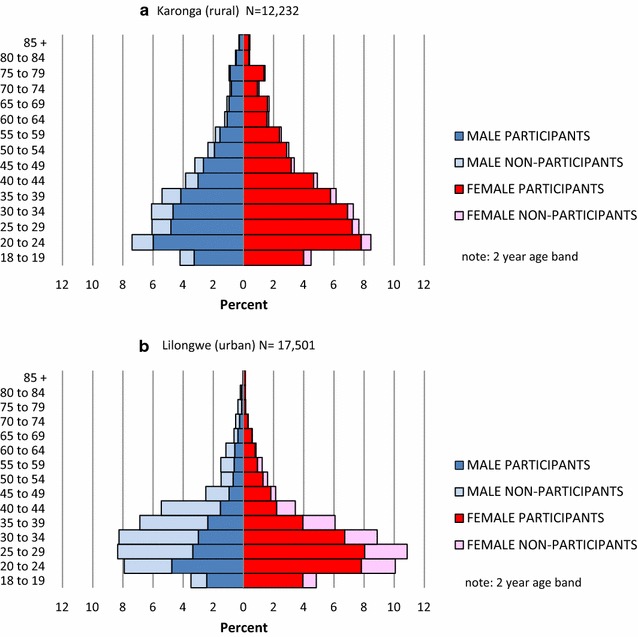
Population structure and participation rates in** a** rural and** b** urban areas by age and sex group. Note: Age distribution of missed/refused by age in urban area is based on a sample of three enumeration areas where approximate ages were determined by interview of informants

The age and sex distributions of the total population in the rural area were estimated by combining data from individuals that participated in the current survey and known ages of missed individuals from available DSS data. In the urban area, the age distribution of missed individuals was estimated from specially sampled urban enumeration areas (data collected from household informants), and combined with survey participants data to compute total urban population distribution (Fig. 5). Precise comparison cannot be made as missing individuals in the urban area only had estimated ages, but overall the urban adult population was younger; median age for males (approximately 32 vs. 34 years) and median age for females (approximately 30 vs. 35 years) and had a lower female to male ratio (1.05:1) than in the rural area (1.2:1).

### Distribution and characteristics of non-participants

The lowest overall participation rates were in urban males (43.6 %) followed by urban females (75.6 %) then rural males (80.3 %) and females (93.6 %). Within urban males the age group 40–44 years (based on estimated ages of those not participating) had the lowest participation rate (28 %). Of those who participated in the survey, urban males were also least likely to accept venepuncture or HIV testing.

### Differences in zone participation rates

283 (4.6 %) of rural households did not participate at all (thus no household level data were available); 196 (68.6 %) of these were households with only a single adult. In the urban area 1084 (16.4 %) of households did not participate at all, 385 (35.5 %) were single adult households and 499 (46.0 %) had two adult members.

Direct comparison of socio-economic status between participants and non-participants across the two sites was not possible as there was no data on urban non-participants. The proportion of people missed varied from 5.5 to 16 % by study zone in the rural area. The two areas with the lowest proportion missed were both fishing communities. The proportion of people missed varied from 23 to 37 % by study zone in the urban site. The enumeration areas with the highest proportion missed were those with seasonal workers at the tobacco processing plants and areas with higher proportion of civil servants and wealthier individuals.

### Mobility in urban population

**Fig. 6:**
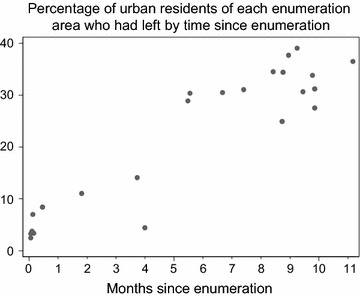
Percentage of urban residents who had left since enumeration over time, by enumeration area

The mobility of the rural population in and out of the DSS is well-described [22] and household movements within the DSS are also well captured. Within the new urban study site, the variable delay between enumeration and survey capture gave an opportunity to measure the rate of movement there, although movements within the area cannot currently be distinguished from those moving outside the area. Those who had left their household between enumeration and survey varied from 3.2 to 38.1 % by enumeration area, which was linearly related to the time between enumeration and survey (Fig. 6). Of those who did participate, 15 % reported they had been resident in that property for less than 1 year. Younger people, females, and poorer people were significantly overrepresented in this group.

### Linkage to health facility data

The study identification number system and referral system worked well, with 100 % identification and linkage of patients in clinic to survey data in both rural and urban site. The use of the health service electronic identity numbers in the urban area was less useful with only 1809 (10 %) of the participants being able, or willing, to provide a verifiable number when visited at their household.

### Outcomes by ease of recruitment and mobility

To explore the importance of the lower participation rates and higher mobility, preliminary analyses were conducted to assess whether ease of recruitment (categorised as either found on first or subsequent visit), whether recruited on a weekday or weekend (urban area only) and duration of residence more than or less than 1 year (urban area), was associated with outcomes and risk factors (Table 1).

**Table 1:** Odds ratios for the association between ease of recruitment and mobility in and risk factors and outcomes

	Category	Crude OR (95 % CI)	Age group and sex adjusted OR (95 % CI) ***p < 0.05
Comparing those recruited on first visit to subsequent visit			
Diabetes			
	1 visit	Ref	Ref
	2/3 visit	1.19 (0.89–1.61)	1.14 (0.84–1.55)
Hypertension			
	1 visit	Ref	Ref
	2/3 visit	1.00 (0.88–1.15)	0.93 (0.79–1.08)
Overweight (BMI ≥ 25)			
	1 visit	Ref	Ref
	2/3 visit	1.07 (0.97– 1.19)	1.21 (1.08–1.34)***
Alcohol user			
	1 visit	Ref	Ref
	2/3 visit	1.47 (1.31–1.65)	1.15 (1.01–1.30)***
Smoker			
	1 visit	Ref	Ref
	2/3 visit	1.07 (0.83–1.38)	0.72 (0.56–0.94)***
Top two wealth quintiles			
	1 visit	Ref	Ref
	2/3 visit	1.22 (1.10–1.35)	1.21 (1.10–1.34)***
Weekend recruitment (urban site only)			
Diabetes			
	Mon–Fri	Ref	Ref
	Sat–Sun	1.05 (0.78–1.42)	0.99 (0.73–1.35)
Hypertension			
	Mon–Fri	Ref	Ref
	Sat–Sun	0.94 (0.82–1.07)	0.85 (0.74–0.99)***
Overweight			
	Mon–Fri	Ref	Ref
	Sat–Sun	0.96 (0.87–1.05)	1.09 (0.98–1.21)
Alcohol user			
	Mon–Fri	Ref	Ref
	Sat–Sun	1.53 (1.37– 1.71)	1.20 (1.06–1.36)***
Smoker			
	Mon–Fri	Ref	Ref
	Sat–Sun	1.01 (0.78–1.29)	0.67 (0.52–0.87)***
Top two wealth quintiles			
	Mon–Fri	Ref	Ref
	Sat–Sun	1.25 (1.13–1.37)	1.24 (1.12–1.36)***
Permanence of residence (urban site only)			
Diabetes			
	≥1 year	Ref	Ref
	<1 year	0.42 (0.26–0.69)	0.75 (0.45–1.22)
Hypertension			
	≥1 year	Ref	Ref
	<1 year	0.57 (0.45–0.65)	0.85 (0.70–1.05)
Overweight			
	≥1 year	Ref	Ref
	<1 year	0.67 (0.60–0.76)	0.76 (0.67–0.86)***
Alcohol user			
	≥1 year	Ref	Ref
	<1 year	0.75 (0.65–0.87)	0.80 (0.68–0.95)***
Smoker			
	≥1 year	Ref	Ref
	<1 year	0.76 (0.55–1.05)	0.96 (0.69–1.35)
Top two wealth quintiles			
	≥1 year	Ref	Ref
	<1 year	0.79 (0.70–0.88)	0.79 (0.70–0.88)***

Adjusting for age and sex, those found on the second or third visit compared to the first visit were more likely to be overweight, current alcohol users and in the top two wealth quintiles. They were less likely to be current smokers. Those surveyed at the weekends were more likely to in the upper wealth bracket and alcohol users, and less likely to be smokers or be hypertensive. People who had been resident in their current property for less than a year were less likely to be overweight or to use alcohol than more permanent residents. These associations remained significant after adjusting for wealth quintile.

## Discussion

Few studies have been population representative and large or detailed enough to adequately describe the emerging cardio-metabolic risk in SSA. The sampling of diverse populations in this large study makes the results relevant to most settings in low-income SSA. The success in designing and implementing this study in rural and urban Malawi has been facilitated by a number of critical factors. Among these was the ability to leverage existing investment in infectious disease research and an established DSS, as a platform to expand into a new programme of research on NCDs. Dedicated funding from the Wellcome Trust allowed development of a focused, but comprehensive, research strategy and infrastructure, including establishing a new urban study site. Early engagement with key stakeholders including policy makers and community leaders ensured alignment with local priorities and, consequently, a high level of support.

Robust protocols and procedures were developed, which have been harmonised across the study sites. This, in addition to the detailed training will ensure generation of the much needed reliable data to inform policy on NCDs and their risk factors in SSA. Many studies have not applied the full rigour to procedures and measurements (e.g. repeated resting blood pressure measurements or formal laboratory glucose assays on fasting participants) [33], which has resulted in inaccurate estimates of prevalence of these conditions.

Another strength of the approach has been to include all adults (≥18 years) to describe accurately the total burden of disease, including the most elderly in whom the highest rates of adverse events occurs and the youngest where the opportunity for prevention of poor health outcomes exists. The detailed nature of the profiling, including the large sample sizes, will facilitate detailed studies of the associations between different modifiable risk factors, including interactions with HIV and ART.

As in the rural DSS, in the urban area great care is being taken in establishing robust linkage systems, such as assigning unique study numbers and documenting spousal and household linkages. These links will enable investigations into the extent to which modifiable risk factors are shared and which will be crucial in identifying high risk groups and designing targeted interventions.

The quick feedback and referral systems, and support (infrastructure, drug supply and staff time) to clinical services has engendered confidence and buy-in from the community to participate, because they recognise that these chronic NCDs can be appropriately managed locally.

## Challenges and lessons learnt

A major challenge has been implementing this ambitious total-adult population survey in an African city with a mobile population and a new study site. The participation rates in Karonga, where the community has longstanding, mutually beneficial engagement with the research group, where individuals are easily found around the home during the day, and where the opportunity to access screening for any condition from other agencies is limited; were excellent. The research team is still building a relationship with urban population, which in itself is more challenging, as the community may be less cohesive and there are high levels of external employment, and more leisure activities away from the household, making establishment of contact more difficult.

The participation rates in the urban area, particularly amongst working males will inevitably result in some selection bias that may either inflate or attenuate the estimates—for example, if less healthy individuals who do not have the capacity to work are more likely to be captured, or, alternatively if the income associated with working males allowed them to engage in less healthy lifestyles. Detailed analyses will be able to impute missing data to some extent but the reality is that the ‘missingness’ is likely to be non-random. This can be demonstrated already, in that those who are harder to find, but who eventually participate, already have significantly different patterns of risk factors and outcomes. It emphasises the importance of flexible study working hours and repeat visits, but also highlights the need to employ innovative strategies to adequately capture the working population. Increasingly, many among this group have access to occupational health schemes at the workplace and/or private medical insurance schemes, both of which are beginning to offer opportunities for hypertension and diabetes screening/care services—reducing the attraction to participate in the survey. For this study ethical approval to engage employers to gain access to individuals in the workplace was not sought, but clearly future interventions will require engagement with a broad spectrum of agencies including occupational health services, private and insurance based health care systems as well as MoH facilities.

As there is intention to follow-up prevalent cases of conditions such as hypertension and diabetes to understand disease progression and test intervention, urban mobility creates another challenge. Further analysis will be required to fully understand these population dynamics to inform design of potential tracking mechanisms and other interventions.

Patients identified with suspected hypertension or diabetes were referred to primary health care clinics in both rural and urban sites, where they are given lifestyle advice and pharmaceutical management initiated as per the MoH treatment protocol. The clinical service is highly integrated to the government settings and close to real life. A major challenge faced was clinic drug supply (both in terms of availability of the spectrum of drugs required to treat hypertension and diabetes of varying levels of intractability and also continuity of supply, even of the basic drugs). We relied largely on external donations. Meeting the increased demand for services generated by enhanced screening and diagnosis of hypertension and diabetes; and mitigating the growing burden of hypertension and diabetes, will require the support and financial commitment of national and international agencies.

## Conclusion

Addressing the public health knowledge-gap around chronic NCD in low-income settings is challenging, in part, due to lack of reliable data on the drivers, burden and distribution (including hard-to-reach populations) of conditions in these settings. With the appropriate support and engagement of community, it is possible to get good participation in most population groups and to generate high quality data, although recruitment in urban males is challenging. This study is representative of rural and urban populations in Malawi and will be crucial in informing national and regional policy in this emerging area.

## Abbreviations

ART: antiretroviral therapy
CVD: cardiovascular disease
DSS: demographic surveillance site
GPS: global positioning system
HIV: human immunodeficiency virus
KPS: Karonga Prevention Study
MEIRU: Malawi Epidemiology and Intervention Research Unit
MoH: Ministry of Health
NCD: non-communicable diseases
RSA: Republic of South Africa
SSA: sub-Saharan Africa
WHO: World Health Organisation
